# Adsorption characteristics of a clinoptilolite-rich zeolite compound for Sr and Cs

**DOI:** 10.1007/s10967-018-6096-6

**Published:** 2018-08-22

**Authors:** Johannes H. Sterba, Hannes Sperrer, Florian Wallenko, Jan M. Welch

**Affiliations:** Atominstitut, TU Wien, Stadionallee 2, 1020 Vienna, Austria

**Keywords:** Zeolite, Clinoptilolite, Strontium, Cesium, Ion exchange

## Abstract

The Sr and Cs adsorption capacities of LithoFill™, LithoGran™ and a competing clinoptilolite containing zeolite product were investigated by radioanalytical methods (^85^Sr and ^134^Cs gamma spectroscopy). The dependence of adsorption and adsorption rate on physical factors including temperature, available ions and time were assessed. In addition, the reversibility of adsorption under high ionic strength conditions was also examined. In general, cesium is more strongly adsorbed than strontium, adsorption yields are generally independent of temperature (from room temperature to 65 °C) and adsorption is relatively rapid (identical results for 2 or 5 day adsorption times). As expected, increasing the concentration of other ions in solution tends to reduce adsorption of cesium and strontium. In general, Cs adsorption ranges between 54.5 and 45.2 mg/g for LitoFill and LitoGran samples and between 36.9 and 24.4 mg/g for the competing product. For Sr adsorption, ranges are 30–21 mg/g and 7.3–6.7 mg/g respectively, leading to the conclusion that the higher content of clinoptilolite in the LitoFill/LitoGran samples results in better adsorption characteristics.

**Electronic supplementary material:**

The online version of this article (10.1007/s10967-018-6096-6) contains supplementary material, which is available to authorized users.

## Introduction

Zeolites, specifically in the form of clinoptilolite ((Na,K,Ca)_2-3_Al_3_(Al,Si)_2_Si_13_O_36_·12(H_2_O)) are well known as natural inorganic ion-exchangers [1–5] with applications in geochemical [6–8], agricultural [9] and decontamination/waste disposal [10–19] operations. The authors received three samples from the company Lithos Natural who have several zeolite mining operations in Lower Austria. Due to the unusually high content of clinoptilolite (~ 90%) in their zeolite, the company claimed exceptionally high ion adsorption capabilities and was interested in the specific properties of their product with respect to the adsorption of cesium and strontium. For comparison, a zeolite with lower clinoptilolite content (~ 60%) was also provided.

## Experimental

To test the adsorption capabilities of the samples for Sr^2+^ and Cs^+^ ions, 500 mg of sample were mixed with 500 µl of stock solutions of CsCl or Sr(NO_3_)_2_ that contained ^134^Cs or ^85^Sr as tracer (see below). If required, additional ions (NaCl or HCl) were added to the mixture. The sample vials were then filled to 10 ml with distilled water and the zeolite powder suspended in the liquid by vortexing. After the planned adsorption time during which the samples were repeatedly vortexed and kept at the desired temperature, liquid and zeolite were separated by centrifugation. The supernatant liquid was decanted, taking care to decant only clear liquid. The decanted liquid was then weighed and measured on a gamma spectrometer to measure the remaining concentration of Cs or Sr in the liquid for comparison to the initial stock solution. From these measurements, the total absorbed Cs or Sr on the zeolite could be calculated.

### Samples

Four samples were provided. Samples A (Lithofill™ 100T) and B (Lithofill™ 100) were composed of particles of < 125 and < 100 µm, respectively. Sample C (Lithogran™) had a particle size of 0.5–2 mm. The three samples A, B, and C each have a clinoptiolite content of approximately 90%. The fourth sample (D) composed of particles sized similarly to sample A was provided for comparison and had a clinoptiolite content of approximately 60%.

### Stock solutions

Stock solutions were prepared by irradiating a small amount of CsCl (10 mg) or Sr(NO_3_)_2_ (30 mg) at a neutron flux density of approximately 2 × 10^12^ cm^−2^ s^−1^ for 8 h in the dry irradiation tube of the TRIGA Mk II reactor of the Atominstitut. The irradiated powders were then dissolved in distilled water and inactive CsCl or Sr(NO_3_)_2_ was added to reach concentrations of approximately 50 mg/ml. The final cesium stock solution had a Cs concentration of 49.43 mg/ml (372 mmol/L). For the strontium experiments, two stock solutions had to be prepared due to the shorter half-life of ^85^Sr. The two solutions had final Sr concentrations of 61.16 mg/ml (698 mmol/L) and 59.99 mg/ml (685 mmol/L).

### Adsorption times

In an initial experiment, different adsorption times were tested for the adsorption of Cs on the zeolite samples. Adsorption times of 2, 4, 10, and 20 min at room temperature (~ 20 °C) were applied. From these initial experiments, it was clear that adsorption reached saturation after 10 min. This time was then used for all further Cs adsorption experiments.

A similar experiment with the Sr stock solution showed that the adsorption was still increasing after 20 min. Thus, adsorption times of 48 and 120 h were applied. After 48 h, the increase in adsorption was only minimal, so 120 h was considered to be a sufficient time for full adsorption.

### Adsorption temperature

To examine the influence of temperature on the adsorption of Cs and Sr, the samples were kept in a water bath at temperatures of 55–65 °C during the adsorption time. The zeolite was kept in suspension by vortexing while maintaining the desired adsorption temperature in the sample. For the Cs and Sr experiments, adsorption time was kept to 5 min.

### Competing ions

It stands to reason that if ions other than Sr or Cs are available in the solution, adsorption behavior will change. To understand how availability of other ions influences Cs and Sr adsorption, NaCl and, in the case of Cs, HCl was added to the solutions before mixing with the zeolite powder. Concentrations were set to 0.005 and 0.006 mol/L for Na^+^ for the Cs and Sr solutions, respectively. This approximately correlates to typical sea-water concentrations. H^+^ concentrations were set to 0.004 mol/L for the Cs solution.

## Results and discussion

In general, it was observed that the adsorption capacities for the three samples A, B, and C are consistently better than for sample D for both Cs^+^ and Sr^2+^ ions. The adsorption capacity for Cs in samples A and B (small particle size) was about 50 mg of Cs per 1 g of sample. For the larger particles in sample C, the adsorption was only slightly smaller at 45 mg/g. For sample D, adsorption of Cs was about 30 mg/g.

The adsorption capabilities of all products for Sr^2+^ ions were approximately half of the capabilities for Cs^+^ and the adsorption process was much slower. We believe this is due to the monovalent versus divalent nature of the cations.

Detailed results are provided in Tables 1 (Cs) and 2 (Sr) as well as in Figs. 1–6 in the supplemental material. An increase in temperature reduced the time necessary to reach saturation. While saturation was reached for Cs adsorption in samples A–C within minutes already at low temperatures, the lower clinoplilotite content of sample D caused slower uptake of Cs, leading to a measureable increase in adsorption at higher temperatures. A similar situation can be seen for the adsorption of Sr, however on a much slower timescale.

**Table 1:** Cs adsorption on samples under different conditions

Sample	Time (min)	Cs uptake (mg/g)	Err%
A	1	49.1	3*
A	2	54.4	3*
A	5	49.8	2.2
A	10	50.4	3*
B	1	54.5	3*
B	2	51.4	3*
B	5	47.8	4.1
B	10	50.2	3*
C	1	40.1	3*
C	2	43.8	3*
C	5	45.2	6.6
C	10	43.0	3*
D	1	24.4	3*
D	2	25.2	3*
D	5	36.9	17.8
D	10	28.9	3*

Sample	Temperature (°C)	Cs uptake (mg/g)	Err%
A	22	50.4	3*
A	55	48.6	3*
A	65	50.5	3*
B	22	50.1	3*
B	55	46.5	3*
B	65	46.9	3*
C	22	46.5	3*
C	55	41.7	3*
C	65	47.2	3*
D	22	30.6	3*
D	55	36.4	3*
D	65	43.7	3*

Sample	Ion concentration (mol/L)	Cs uptake (mg/g)	Err%
A	0	50.4	3*
A	0.004	46.2	0.7
A	0.005	38.9	3*
B	0	50.1	3*
B	0.004	47.8	0.8
B	0.005	41.2	3*
C	0	46.5	3*
C	0.004	41.9	6.4
C	0.005	31.9	3*
D	0	30.6	3*
D	0.004	24.1	3.2
D	0.005	23.7	3*

Since not all adsorption experiments were repeated, error is estimated from counting statistics at 3% as indicated by “*”. All other errors were calculated as the standard deviation of repeat experiments

**Table 2:** Sr adsorption on samples under different conditions

Sample	Time (h)	Sr uptake (mg/g)	Err%
A	0.08	13	46
A	48	23	3.4
A	120	30	2.8
B	0.08	21	27
B	48	26	4.3
B	120	26	5.9
C	0.08	11	26
C	48	19	3.3
C	120	21	7.4
D	0.08	6.7	22
D	48	6.7	10
D	120	7.3	24

Sample	temperature (°C)	Sr uptake (mg/g)	Err%
A	22	8.1	21
A	60	19	6.2
B	22	17	12
B	60	27	5.1
C	22	9.3	6.2
C	60	15	10
D	22	4.9	16
D	60	6.7	25

Sample	ion concentration (mol/L)	Sr uptake (mg/g)	Err%
A	0	30	2.8
A	0.006	16	1.7
B	0	26	5.9
B	0.006	9.3	29
C	0	21	7.4
C	0.006	7.5	17
D	0	7.3	24
D	0.006	4.1	46

All errors were calculated as the standard deviation of 4 repeat experiments

The experiments with additional, competing ions show that in the case of Cs adsorption, saturation was reached since a significant decrease in Cs adsorption was observed. This indicates that the competing ions may displace the Cs ions. The situation is similar for the uptake of Sr.

## Conclusion and outlook

From the data collected during this study it can be concluded that the major factor influencing for the adsorption of Cs and Sr on zeolites is the content of clinoptilolite. Higher temperature increases the rate of uptake to saturation of the zeolite. Under the conditions used, for Cs, adsorption is very fast (on the order of minutes) whereas for Sr several hours are needed to reach saturation. Also, in general, adsorption capacity for Cs is about double the capacity for Sr. Both, the longer adsorption time as well as the lower adsorption capacity are most probably due to the divalent nature of Sr. Ionic radius and the nature of exchanges sites within the zeolite structure are also likely to influence capacity and adsorption rate. Finally, smaller particle sizes speed adsorption, which might be expected due to the larger surface area available.

Further studies of the Cs and Sr adsorption under the influence of several different concentrations of competing ions, including divalent species, up to similar concentrations as in the stock solution would be useful. While this would most probably not be a case in a practical application as a remediation medium, more detailed information on the selectivity of clinoptilolite could be gained. Furthermore, the adsorption kinetics for the Sr^2+^ ions should be studied further, hopefully gaining insight into the structural properties of the clinoptilolite that enable mono- and divalent ion absorption.

## Electronic supplementary material

Below is the link to the electronic supplementary material.

Supplementary material 1 (PDF 4 kb)

Supplementary material 2 (PDF 4 kb)

Supplementary material 3 (PDF 4 kb)

Supplementary material 4 (PDF 4 kb)

Supplementary material 5 (PDF 4 kb)

Supplementary material 6 (PDF 4 kb)
