# Supplementary figures and images for: Adsorption characteristics of a clinoptilolite-rich zeolite compound for Sr and Cs
Source: J Radioanal Nucl Chem. 2018 Aug 22;318(1):267–70. doi: 10.1007/s10967-018-6096-6 (PMC6182729; doi:10.1007/s10967-018-6096-6)

Absorbed Cs by Time

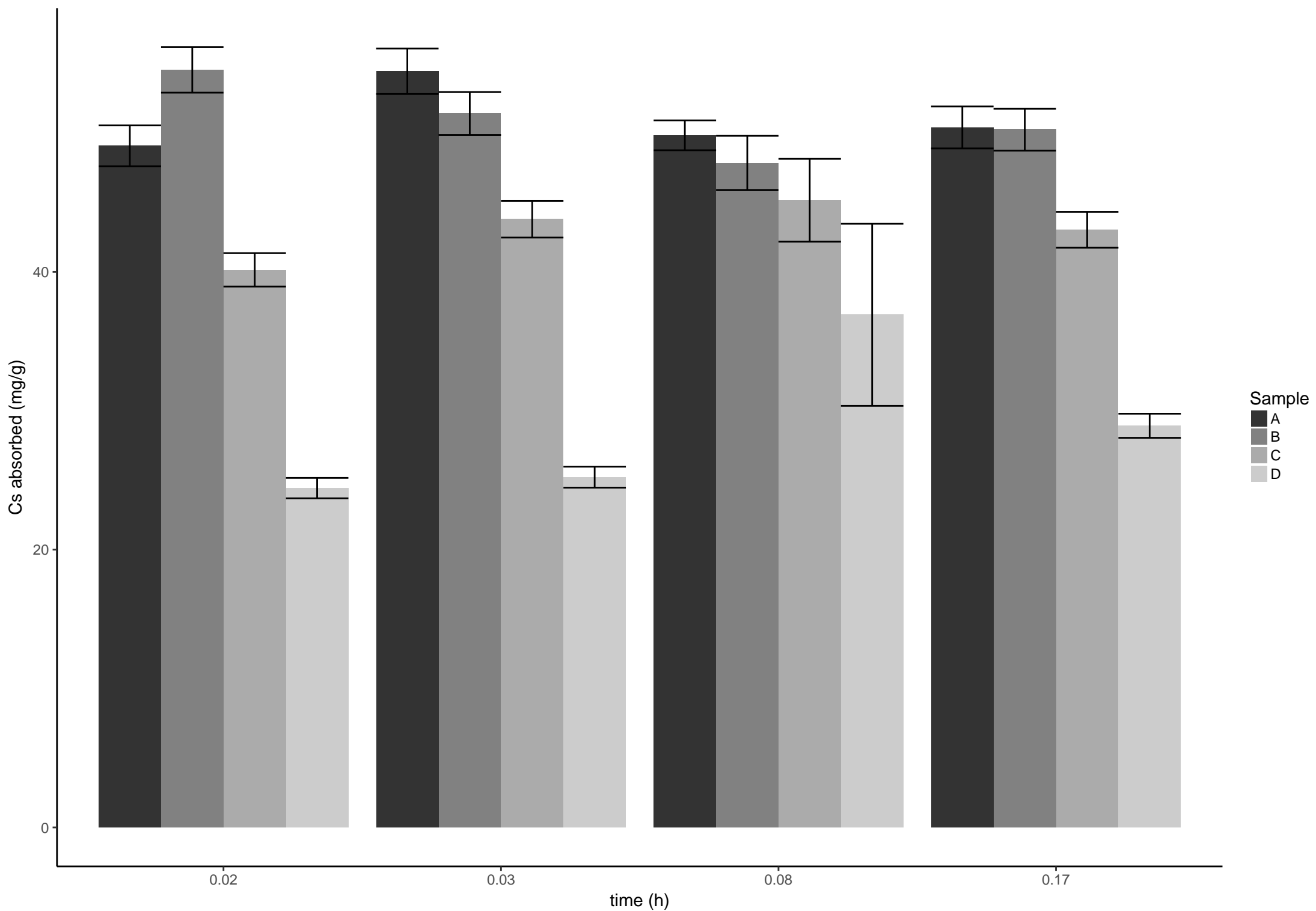

Supplement: Supplementary file 1 — Supplementary material 1 (PDF 4 kb) [file 10967_2018_6096_MOESM1_ESM.pdf]

Absorbed Sr by Time

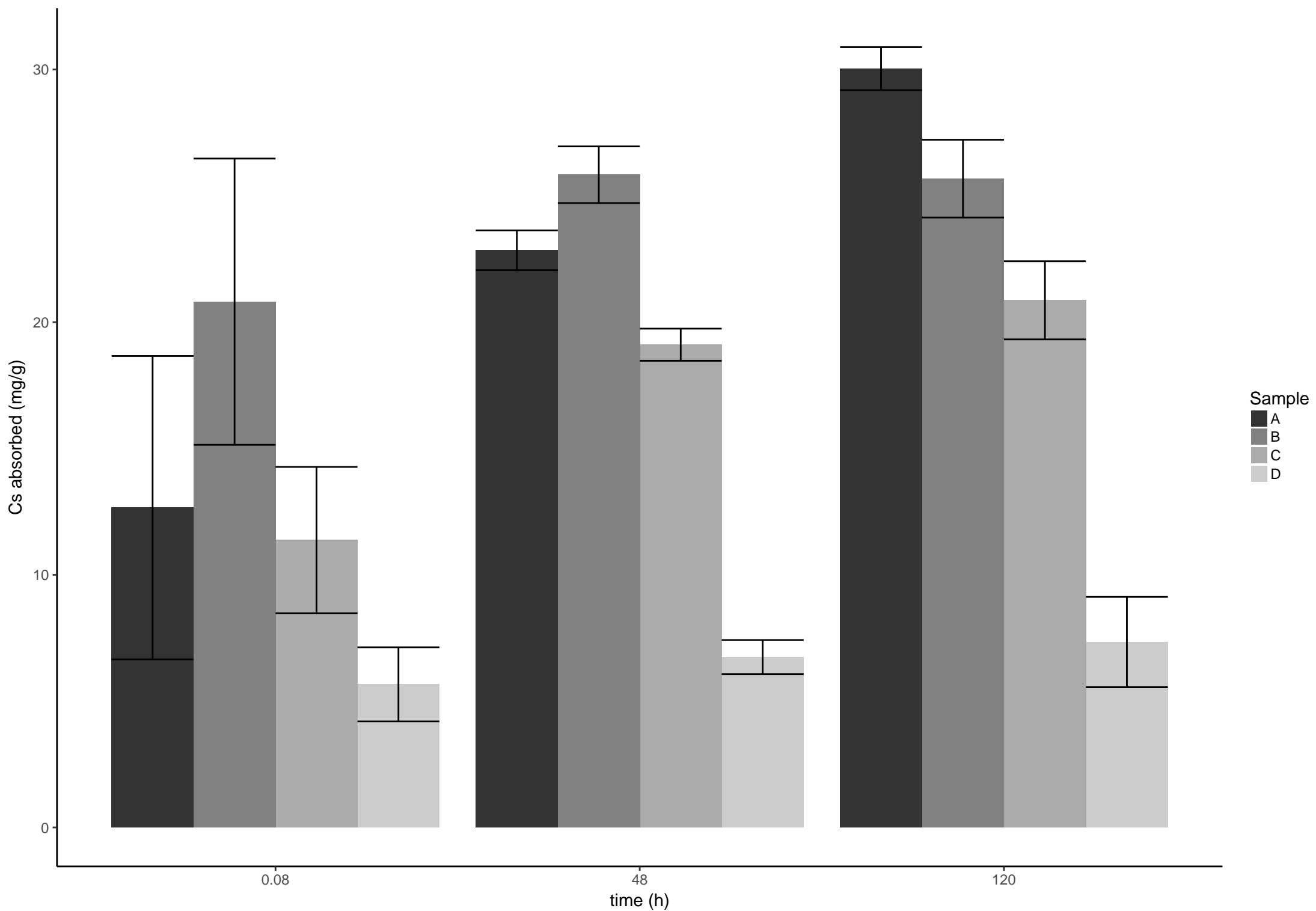

Supplement: Supplementary file 2 — Supplementary material 2 (PDF 4 kb) [file 10967_2018_6096_MOESM2_ESM.pdf]

Absorbed Cs by Temperature

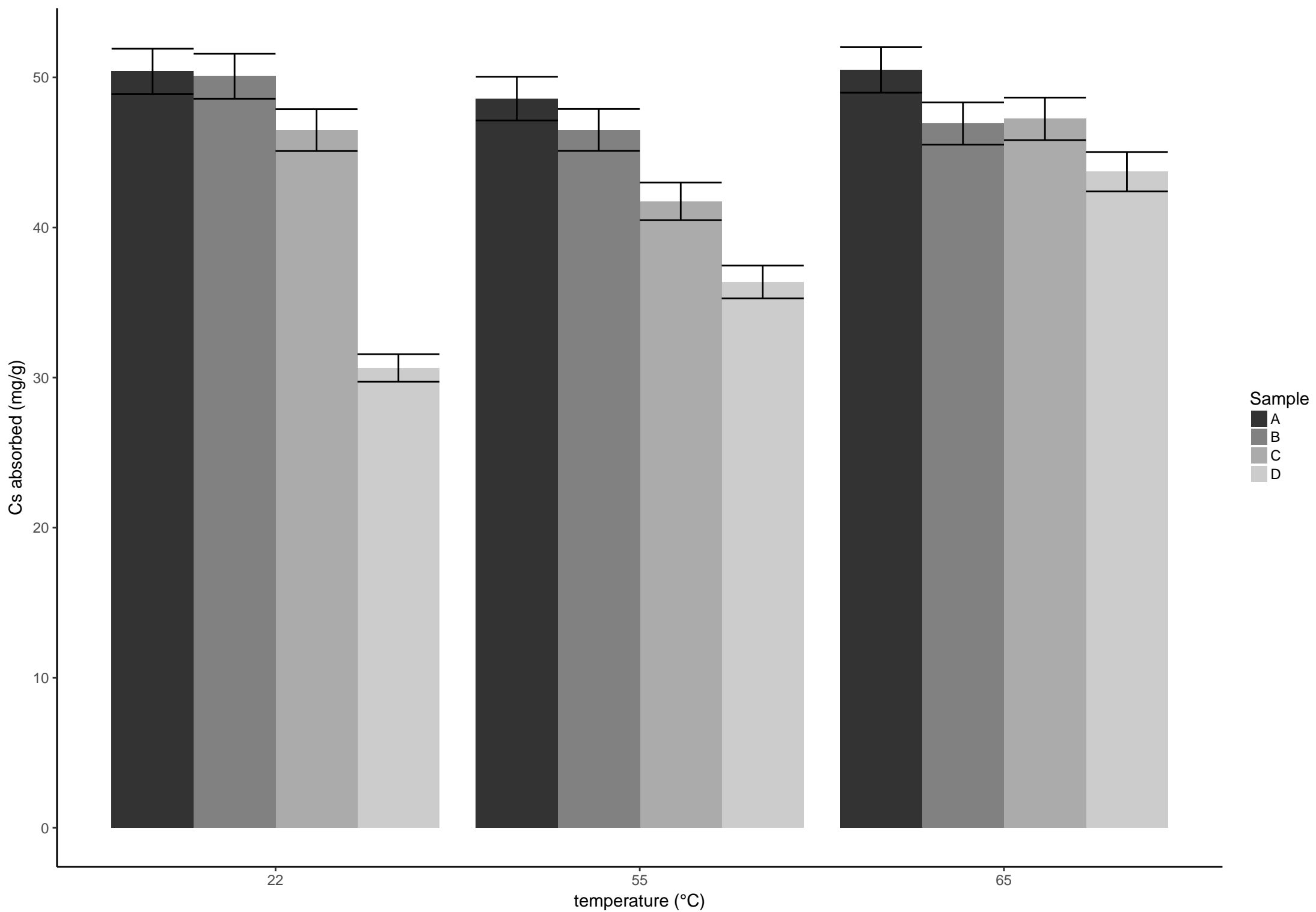

Supplement: Supplementary file 3 — Supplementary material 3 (PDF 4 kb) [file 10967_2018_6096_MOESM3_ESM.pdf]

Absorbed Sr by Temperature (5 mins)

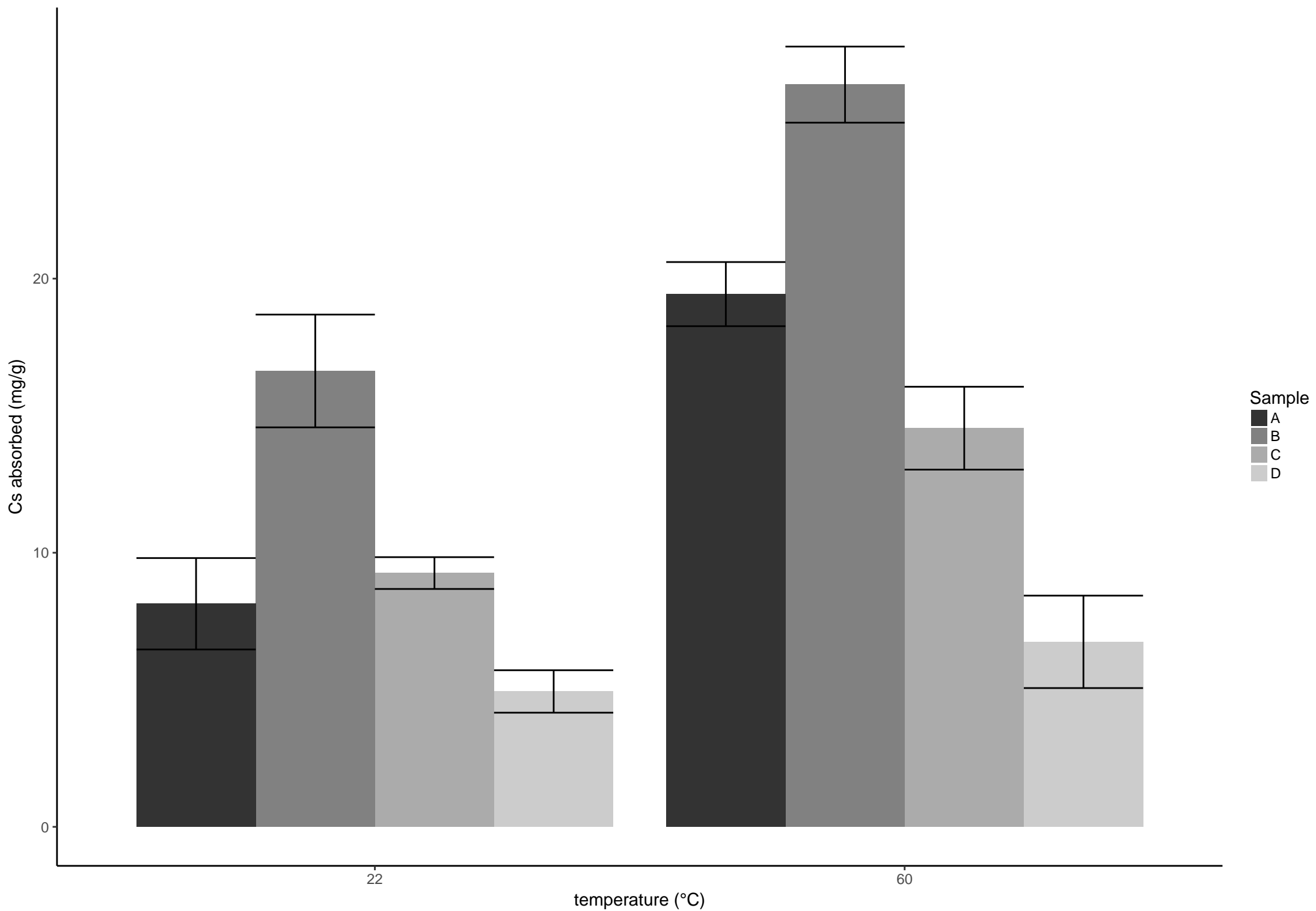

Supplement: Supplementary file 4 — Supplementary material 4 (PDF 4 kb) [file 10967_2018_6096_MOESM4_ESM.pdf]

Absorbed Cs by competing ions

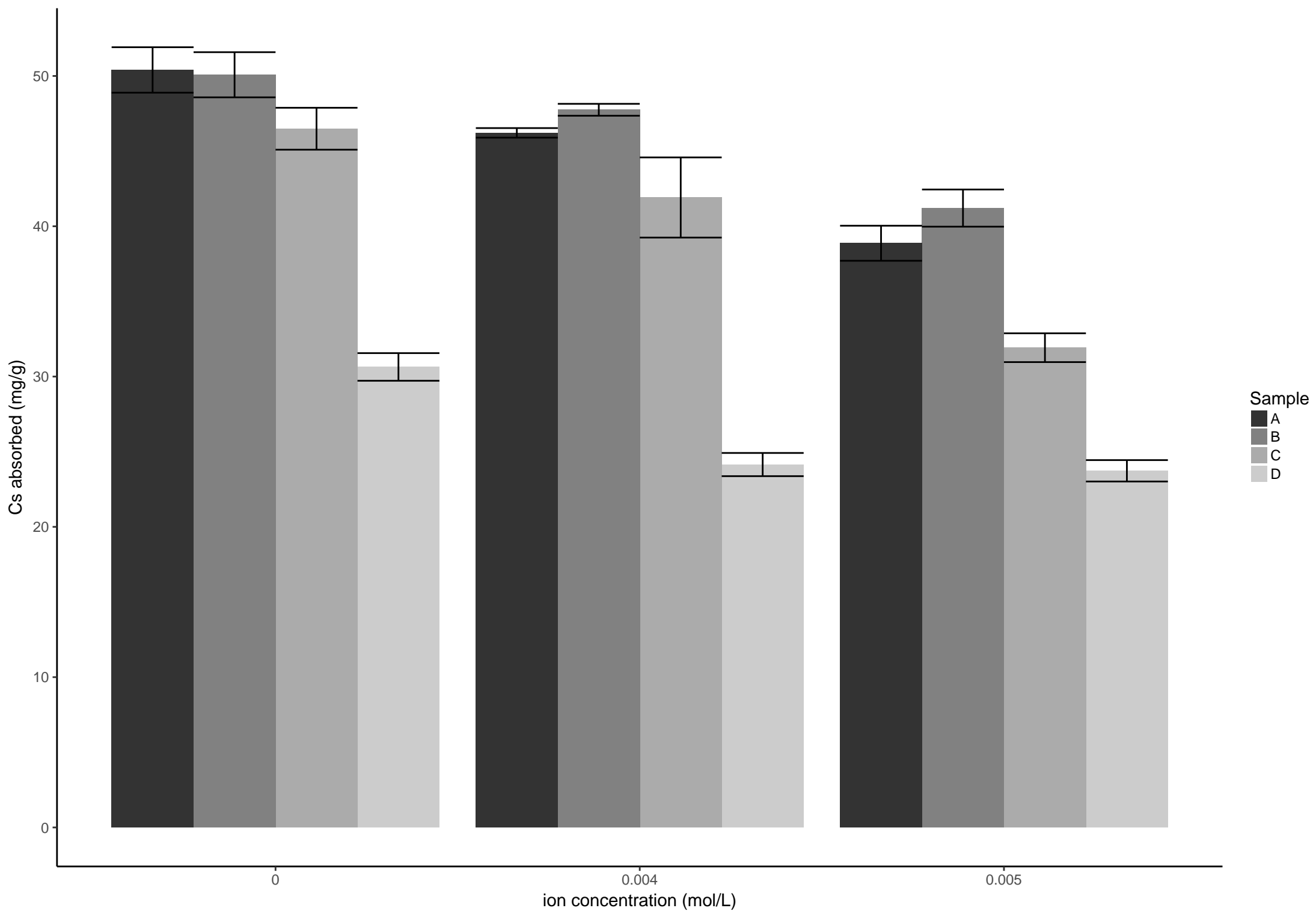

Supplement: Supplementary file 5 — Supplementary material 5 (PDF 4 kb) [file 10967_2018_6096_MOESM5_ESM.pdf]

Absorbed Sr by competing ions (120 hours)

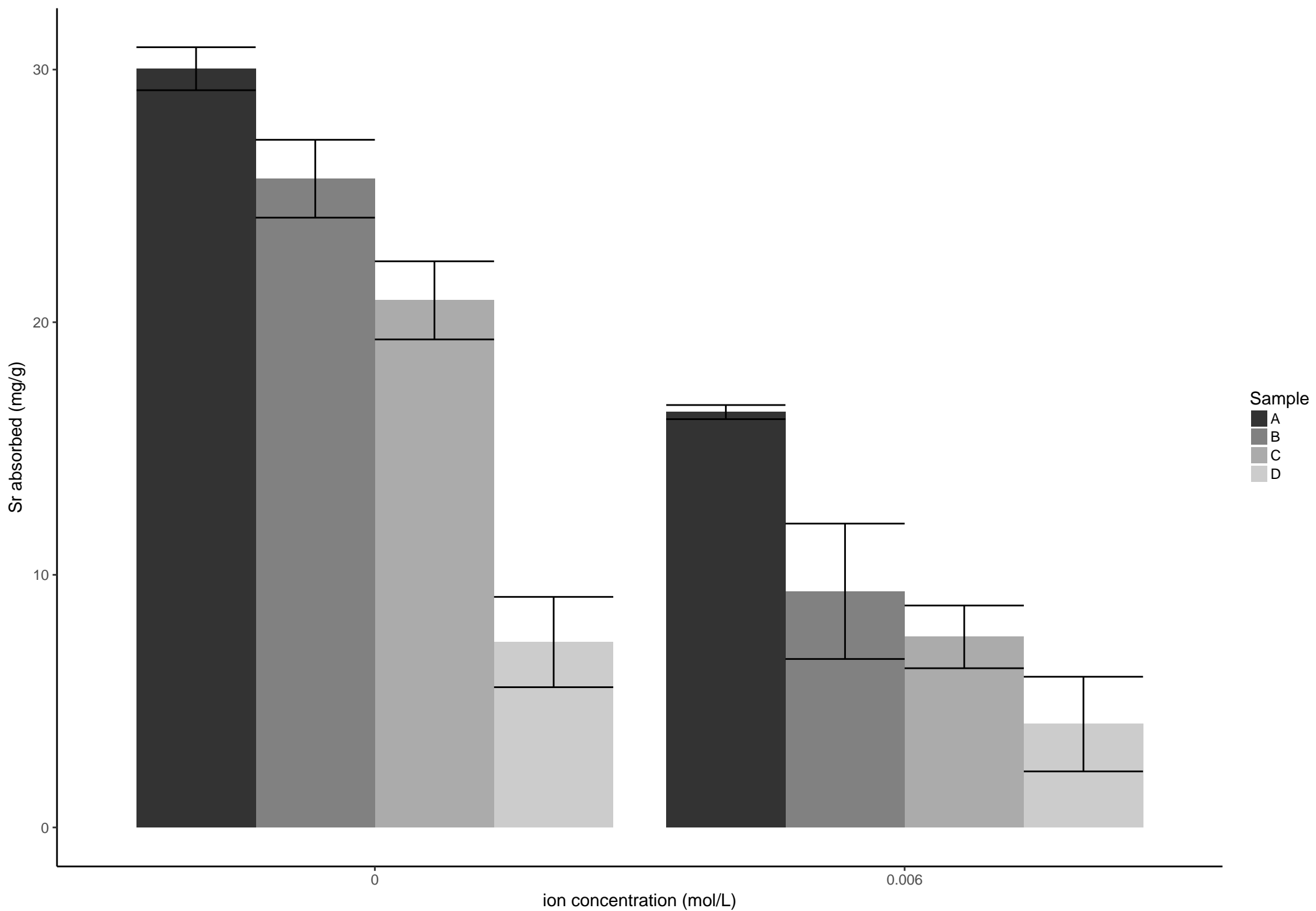

Supplement: Supplementary file 6 — Supplementary material 6 (PDF 4 kb) [file 10967_2018_6096_MOESM6_ESM.pdf]
